# Strengthening the Reporting of Observational Studies in Epidemiology – nutritional epidemiology (STROBE‐nut): An extension of the STROBE statement†

**DOI:** 10.1111/nbu.12217

**Published:** 2016-08-14

**Authors:** C. Lachat, D. Hawwash, M. C. Ocké, C. Berg, E. Forsum, A. Hörnell, C. l. Larsson, E. Sonestedt, E. Wirfält, A. Åkesson, P. Kolsteren, G. Byrnes, W. De Keyzer, J. Van Camp, J. E. Cade, N. Slimani, M. Cevallos, M. Egger, I. Huybrechts

**Affiliations:** ^1^ Department of Food Safety and Food QualityGhent UniversityGhentBelgium; ^2^ Unit of Nutrition and Child HealthInstitute of Tropical MedicineAntwerpBelgium; ^3^ National Institute for Public Health and the EnvironmentBilthovenThe Netherlands; ^4^ Department of Food and Nutrition, and Sport ScienceUniversity of GothenburgGothenburgSweden; ^5^ Department of Clinical and Experimental MedicineLinköping UniversityLinköpingSweden; ^6^ Department of Food and NutritionUmeå UniversityUmeåSweden; ^7^ Department of Clinical Sciences MalmöLund UniversityMalmöSweden; ^8^ Institute of Environmental MedicineKarolinska InstitutetStockholmSweden; ^9^ International Agency for Research on CancerLyonFrance; ^10^ Department of Biosciences and Food SciencesUniversity College GhentGhentBelgium; ^11^ School of Food Science and NutritionUniversity of LeedsLeedsUK; ^12^ Institute of Social and Preventive MedicineUniversity of BernBernSwitzerland; ^13^ Department of Clinical ResearchUniversity of BernBernSwitzerland

**Keywords:** dietary assessment, nutritional epidemiology, reporting guidelines, research methodology

## Abstract

Concerns have been raised about the quality of reporting in nutritional epidemiology. Research reporting guidelines such as the Strengthening the Reporting of Observational Studies in Epidemiology (STROBE) statement can improve quality of reporting in observational studies. Herein, we propose recommendations for reporting nutritional epidemiology and dietary assessment research by extending the STROBE statement into Strengthening the Reporting of Observational Studies in Epidemiology – Nutritional Epidemiology (STROBE‐nut). Recommendations for the reporting of nutritional epidemiology and dietary assessment research were developed following a systematic and consultative process, co‐ordinated by a multidisciplinary group of 21 experts. Consensus on reporting guidelines was reached through a three‐round Delphi consultation process with 53 external experts. In total, 24 recommendations for nutritional epidemiology were added to the STROBE checklist. When used appropriately, reporting guidelines for nutritional epidemiology can contribute to improve reporting of observational studies with a focus on diet and health.

## Introduction

Nutritional epidemiology examines the relationship between diet and health in human populations. The assessment of diet is, however, complex and suffers from considerable measurement error (Box 1). As a consequence, concerns have been raised about epidemiological research regarding diet and human health (Schoenfeld & Ioannidis 2013), and two systematic reviews identified reporting quality as a problem (Gibson *et al*. 2005; Bekkering *et al*. 2008). Furthermore, all but four of the 17 literature reviews performed prior to the fifth revision of the Nordic Nutrition Recommendations (Nordic Council of Ministers 2014) report that problems with a lack of methodological details (*e.g*. recruitment, dropout, compliance, statistical methods and dietary intake assessment) caused lower quality rating or exclusion of papers.

Box 1 **Assessment of dietary intake as an exposure in nutritional epidemiology**

The human diet is the result of interacting food constituents and cultural processes that remain poorly documented. As each food item contains a number of bioactive substances, covariation is common between dietary components. It may be extremely difficult to isolate the specific effect of a single food component. In addition, lifestyle and socio‐economic factors also covary with diet. Because of this complex nature of our diet, dietary and nutritional assessment is prone to particular types of random and systematic errors (bias) that can occur in different ways (*e.g*. through selection and sampling bias, recall bias, interviewer bias, coding bias and day‐to‐day variability in our diet). Clear reporting of nutritional epidemiological research is essential to ensure correct assessment of observational studies, as illustrated with the controversy surrounding saturated fats and risk of coronary heart disease (Chowdhury *et al*. 2014).

To date, several methods are available for conducting dietary assessments, though each of them has inherent strengths and limitations. Methods to assess dietary intake include (i) food frequency questionnaires, principally used to assess long‐term average intakes; (ii) 24‐hour recall as a memory‐based short‐term dietary assessment method; (iii) a food diary, which prospectively collects dietary intake data; (iv) diet history; and (v) checklist questions that assess one specific aspect of dietary intake. Tools to assess dietary intake have mostly been paper based in the past. To date, new approaches available include applications such as web‐based tools, mobile phone applications, camera and photographic methods and bar code scanners. Although these approaches are promising, validation information is only limited, and issues regarding measurement error may remain (Illner *et al*. 2012).

Investigating the validity of methods and procedures used in nutritional epidemiology is crucial given the complexity of our diet and the multiple sources of bias that impact the quality of dietary assessments. Sometimes a reliable standard is available against which the validity of a survey method can be assessed. However, advances in nutritional epidemiological research have been constrained by the lack of gold standards against which dietary assessment tools can be validated. Only a few ideal reference measures are currently available (*i.e*. stable isotopes like doubly labelled water for energy intake, the recovery biomarker 24‐hour urinary nitrogen excretion for protein intake and 24‐hour urinary potassium excretion for potassium intake). For practical reasons, however, non‐ideal reference measures such as 24‐hour dietary recalls or food records are often used.

Biomarkers are often used as objective and/or complementary measures of dietary intake. They include (i) recovery markers, which provide an estimate of absolute nutrient intake over a fixed time period (*e.g*. urinary nitrogen or protein); (ii) predictive biomarkers, which have a lower overall recovery (*e.g*. urinary fructose, sucrose and dietary sugars); and (iii) concentration biomarkers, which do not reflect intake but are correlated with intake (*e.g*. plasma vitamin C, carotenoids and vitamin E). However, objective measures to assess dietary intake are not without limitations and may provide only a partial evaluation of the complexity of the human diet.

In conclusion, as different dietary assessment methods suffer from specific measurement error, a careful description of the method used and its limitations is essential to allow correct interpretation of the findings.

Readers of poorly reported studies may reach erroneous conclusions and inappropriately implement the findings in clinical settings, population interventions or other research (Glasziou *et al*. 2014). The need to ensure clear, transparent and useful reports in health research led to important reporting initiatives such as the Strengthening the Reporting of Observational Studies in Epidemiology (STROBE) statement (Vandenbroucke *et al*. 2007). The STROBE statement is an evidence‐based minimum set of recommendations for reporting of observational studies. It consists of a set of 22 items to report cohort studies, case–control studies and cross‐sectional studies. The use of the recommendations has influenced the style of reporting (Fung *et al*. 2009). However, there is evidence of misuse (da Costa *et al*. 2011). The STROBE recommendations should not be considered as prescriptions for designing or conducting studies or as an instrument to evaluate the quality of observational research. These reporting guidelines rather provide guidance on how to improve completeness and transparency of research reports.

Herein, we propose recommendations for reporting nutritional epidemiology and dietary assessment research by extending the STROBE statement into the STROBE Extension for Nutritional Epidemiology (STROBE‐nut). A forthcoming study will explain and elaborate the STROBE‐nut recommendations to enhance clarity and facilitate understanding of the guidelines.

## Methodology

The STROBE‐nut checklist and recommendations were developed following recommended procedures (Moher *et al*. 2010). Three groups of researchers that independently and concurrently had developed initiatives with similar aims joined forces. A steering group of 21 members consisting of individuals, including journal editors, with expertise in nutritional epidemiology, dietary assessment, dietetics and medical ethics, co‐ordinated the study.

**Figure 1:**
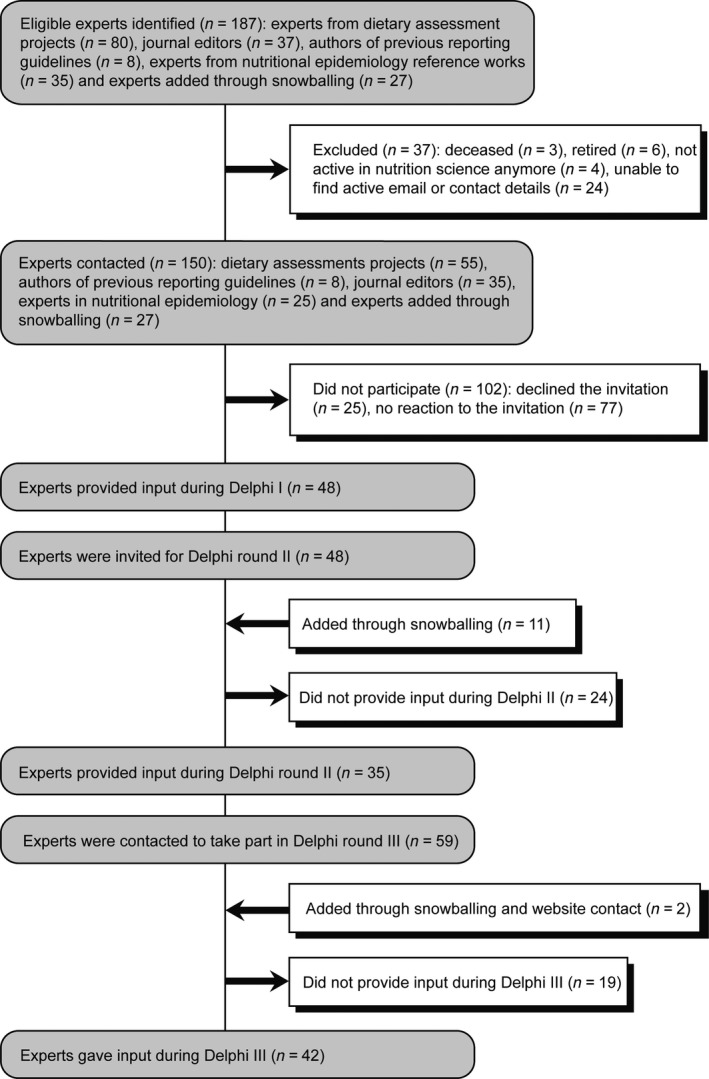
Participants of the Delphi consultation.

A protocol was registered prospectively (Hawwash & Lachat 2014). Experts (*i.e*. methodologists, journal editors, statisticians, epidemiologists and content experts) were identified from relevant methodological projects and reference documents, and provided the recommendations of the checklist (Box 2). Snowballing and announcements via the STROBE‐nut website (www.strobe-nut.org) were used to raise awareness and involve additional participants. A total of 150 experts were invited, of whom 53 provided input during at least one consultation round (Fig. 1).

Box 2 **Recruitment strategy of experts for the Delphi rounds**

- Journal editors: Editors‐in‐chief of the first 50% of journals in the subject category ‘Nutrition and dietetics’ sorted by impact factor in the Web of Science in 2012 were identified. Editors were grouped per publisher and contacted accordingly.
- Methodologists, nutritional epidemiologists and the content experts:
  ○ Corresponding authors of previous initiatives to improve the reporting of dietary assessments or nutritional epidemiology were invited (Nelson *et al*. 1993; FAO 2010; Welch *et al*. 2011; Burrows *et al*. 2012; Faber *et al*. 2013). If they were unwilling to participate, then the last author was contacted. We also contacted the editors and authors of two reference books in nutritional epidemiology (Nelson & Margetts 1997; Willett 2013).
  ○ Work package leaders and principal investigators of methodological projects in dietary assessment [*i.e. European Food Consumption Validation* (*EFCOVAL*), *European Food Consumption Survey Method* (*EFCOSUM*), *Observing Protein and Energy Nutrition* (*OPEN*) study, *EU MENU – Harmonising Data Collection on Food Consumption across Europe, Pilot Study for the Assessment of Nutrient Intake and Food Consumption among Kids in Europe* (*PANCAKE*) and *Africa's Study on Physical Activity and Dietary Assessment Methods* (*AS‐PADAM*) and networks in nutrition epidemiology, including the *African Nutrition Epidemiology Conference* (*ANEC*) and the *Swedish Network in Epidemiology and Nutrition* (*NEON*)].

This study was approved by the Ethics Committee of Ghent University, and experts provided informed consent for the study. Data collection started on 12 February 2014.

## STROBE‐nut Recommendations

A formal Delphi process was used to ensure broad consultation and increase the involvement of editors and researchers with various expertises and experiences. Consensus was reached through a three‐round process (Table S1). As proposed previously (Sinha *et al*. 2011), items were retained when >70% and >80% of agreement were obtained during the second and third consultation round, respectively. During three face‐to‐face meetings, members of the steering group discussed the input received and prepared new versions that were circulated until consensus was reached. Disagreement was discussed and added to the meeting minutes.

The response rates were 32.0% (48/150), 59.3% (35/59) and 68.9% (42/61), respectively, during the three Delphi rounds. After the second and third Delphi round, six and two items, respectively, were removed, as insufficient consensus was reached. Two items (nut‐22.1 and nut‐22.2) were added to STROBE in line with present recommendations (Glasziou *et al*. 2014; International Committee of Medical Journal Editors 2015). There was an average agreement of 97.1% (standard deviation 3.6%) to retain a final list of 24 STROBE‐nut recommendations (Table 1). When no specific STROBE‐nut item is listed in Table 1, this indicates that the original STROBE item alone was considered sufficient. Below follows a description of the specific STROBE‐nut items.

**Table 1:** STROBE‐nut: An extension of the STROBE statement for nutritional epidemiology

Item	Item number	STROBE recommendations	Extension for Nutritional Epidemiology Studies (STROBE‐nut)
Title and abstract	1	(a) Indicate the study's design with a commonly used term in the title or the abstract	**nut‐1.** State the dietary/nutritional assessment method(s) used in the title, abstract or keywords
		(b) Provide in the abstract an informative and balanced summary of what was done and what was found	
Introduction			
Background Rationale	2	Explain the scientific background and rationale for the investigation being reported	
Objectives	3	State specific objectives, including any pre‐specified hypotheses	
Methods			
Study design	4	Present key elements of study design early in the study	
Settings	5	Describe the setting, locations and relevant dates, including periods of recruitment, exposure, follow‐up and data collection	**nut‐5.** Describe any characteristics of the study settings that might affect the dietary intake or nutritional status of the participants, if applicable
Participants	6	(a) Cohort study – give the eligibility criteria and the sources and methods of selection of participants. Describe methods of follow‐up	**nut‐6.** Report particular dietary, physiological or nutritional characteristics that were considered when selecting the target population
		Case–control study – give the eligibility criteria and the sources and methods of case ascertainment and control selection. Give the rationale for the choice of cases and controls	
		Cross‐sectional study – give the eligibility criteria and the sources and methods of selection of participants	
		(b) Cohort study – for matched studies, give matching criteria and number of exposed and unexposed	
		Case–control study – for matched studies, give matching criteria and the number of controls per case	
Variables	7	Clearly define all outcomes, exposures, predictors, potential confounders and effect modifiers. Give diagnostic criteria, if applicable	**nut‐7.1.** Clearly define foods, food groups, nutrients or other food components
			**nut‐7.2.** When using dietary patterns or indices, describe the methods to obtain them and their nutritional properties
Data sources – measurements	8	For each variable of interest, give sources of data and details of methods of assessment (measurement). Describe comparability of assessment methods if there is more than one group	**nut‐8.1.** Describe the dietary assessment method(s) (*e.g*. portion size estimation, number of days and items recorded), how it was developed and administered and how quality was assured. Report if and how supplement intake was assessed
			**nut‐8.2.** Describe and justify food composition data used. Explain the procedure to match food composition with consumption data. Describe the use of conversion factors, if applicable
			**nut‐8.3.** Describe the nutrient requirements, recommendations or dietary guidelines and the evaluation approach used to compare intake with the dietary reference values, if applicable
			**nut‐8.4.** When using nutritional biomarkers, additionally use the STROBE Extension for Molecular Epidemiology (STROBE‐ME). Report the type of biomarkers used and their usefulness as dietary exposure markers
			**nut‐8.5.** Describe the assessment of non‐dietary data (*e.g*. nutritional status and influencing factors) and timing of the assessment of these variables in relation to dietary assessment
			**nut‐8.6.** Report on the validity of the dietary or nutritional assessment methods and any internal or external validation used in the study, if applicable
Bias	9	Describe any efforts to address potential sources of bias	**nut‐9.** Report how bias in dietary or nutritional assessment was addressed (*e.g*. misreporting, changes in habits as a result of being measured or data imputation from other sources)
Study size	10	Explain how the study size was arrived at	
Quantitative variables	11	Explain how quantitative variables were handled in the analyses. If applicable, describe which groupings were chosen and why	**nut‐11.** Explain the categorisation of dietary/nutritional data (*e.g*. use of N‐tiles and handling of non‐consumers) and the choice of reference category, if applicable
Statistical methods	12	(a) Describe all statistical methods, including those used to control for confounding. (b) Describe any methods used to examine subgroups and interactions. (c) Explain how missing data were addressed. (d) Cohort study – if applicable, explain how loss to follow‐up was addressed. Case–control study – if applicable, explain how matching of cases and controls was addressed. Cross‐sectional study – if applicable, describe analytical methods taking account of sampling strategy. (e) Describe any sensitivity analyses	**nut‐12.1.** Describe any statistical method used to combine dietary or nutritional data, if applicable
			**nut‐12.2.** Describe and justify the method for energy adjustments, intake modelling and use of weighting factors, if applicable
			**nut‐12.3.** Report any adjustments for measurement error (*i.e*. from a validity or calibration study)
Results			
Participants	13	(a) Report the numbers of individuals at each stage of the study (*e.g*. numbers potentially eligible, examined for eligibility, confirmed eligible, included in the study, completing follow‐up and analysed). (b) Give reasons for non‐participation at each stage. (c) Consider use of a flow diagram	**nut‐13.** Report the number of individuals excluded based on missing, incomplete or implausible dietary/nutritional data
Descriptive data	14	(a) Give characteristics of study participants (*e.g*. demographic, clinical and social) and information on exposures and potential confounders. (b) Indicate the number of participants with missing data for each variable of interest. (c) Cohort study – summarise follow‐up time (*e.g*. average and total amount)	**nut‐14.** Give the distribution of participant characteristics across the exposure variables if applicable. Specify if food consumption of total population or consumers only were used to obtain results
Outcome data	15	Cohort study – report numbers of outcome events or summary measures over time. Case–control study – report numbers in each exposure category or summary measures of exposure. Cross‐sectional study – report numbers of outcome events or summary measures	
Main results	16	(a) Give unadjusted estimates and, if applicable, confounder‐adjusted estimates and their precision (*e.g*. 95% confidence interval). Make clear which confounders were adjusted for and why they were included. (b) Report category boundaries when continuous variables were categorised. (c) If relevant, consider translating estimates of relative risk into absolute risk for a meaningful time period	**nut‐16.** Specify if nutrient intakes are reported with or without inclusion of dietary supplement intake, if applicable
Other analyses	17	Report other analyses done (*e.g*. analyses of subgroups and interactions and sensitivity analyses.	**nut‐17.** Report any sensitivity analysis (*e.g*. exclusion of misreporters or outliers) and data imputation, if applicable
Discussion			
Key results	18	Summarise key results with reference to study objectives	
Limitation	19	Discuss limitations of the study, taking into account sources of potential bias or imprecision. Discuss both direction and magnitude of any potential bias	**nut‐19.** Describe the main limitations of the data sources and assessment methods used and implications for the interpretation of the findings
Interpretation	20	Give a cautious overall interpretation of results considering objectives, limitations, multiplicity of analyses, results from similar studies and other relevant evidence	**nut‐20.** Report the nutritional relevance of the findings, given the complexity of diet or nutrition as an exposure
Generalisability	21	Discuss the generalisability (external validity) of the study results	
Other information			
Funding	22	Give the source of funding and the role of the funders for the present study and, if applicable, for the original study on which the present article is based	
Ethics			**nut‐22.1.** Describe the procedure for consent and study approval from ethics committee(s)
Supplementary material			**nut‐22.2.** Provide data collection tools and data as online material or explain how they can be accessed

**nut‐1. State the dietary/nutritional assessment method(s) used in the title, abstract or keywords**

Referring to the assessment methods in the title, abstract or keywords will facilitate correct indexing and retrieval of studies. The title and abstract are the parts of papers most read, and their content will influence the reader's decision on further considerations of the paper. Inclusion of nutritional epidemiological terms is particularly necessary when the method is important to interpret the study findings.

**nut‐5. Describe any characteristics of study settings that might affect the dietary intake or nutritional status of the participants, if applicable**

A clear description of the study settings is essential to understand the external conditions that affect the estimation of dietary intake or nutritional status of the participants. Hence, factors that may affect dietary intake, nutritional status or dietary reporting should be carefully described. These factors can, for instance, be location (*e.g*. areas or institutions) and time frame of the study (*e.g*. season, festivities or fasting periods).

**nut‐6. Report particular dietary, physiological or nutritional characteristics that were considered when selecting the participants**

Accurate reporting of the characteristics used to include or exclude participants is needed as they may affect the interpretation and generalisability of the findings. Age, gender, dietary habits, physical activity, smoking, body mass index and physiological status (*e.g*. pregnancy or illness) are examples of such characteristics.

**nut‐7.1. Clearly define foods, food groups, nutrients or other food components**

Foods, nutrients and other components should be clearly defined and specified, possibly by using scientific names (*i.e*. chemical form for compounds or taxonomical name for specific plants or animals). In case of complex foods or recipes, ingredients, amounts and preparation methods should be stated when possible. Any aggregation of food or classification of food groups should be defined.

**nut‐7.2. When using dietary patterns or indices, describe the methods to obtain them and their nutritional properties**

The approach and variables used to derive dietary patterns should be described, including if and how energy intake was considered. A rationale for the development of an *a priori* dietary index or score should be given together with an explanation of how the scoring of each component was done and how the various components were combined. The potential and observed range of the score should be given, together with a central measure and distribution.
For exploratory approaches (*e.g*. principal component analyses, factor analyses and cluster analyses), the statistical procedure and software used should be described. The steps and decisions taken to define the dietary patterns should be explained in addition to the nutritional characteristic of each pattern. If hybrid methods such as reduced rank regression are used, also describe the dependent variables.

**nut‐8.1. Describe the dietary assessment method(s) (** ***e.g.*** **portion size estimation, number of days and items recorded), how it was developed and administered and how quality was assured. Report if and how supplement intake was assessed**

Describe the main dietary assessment method (*e.g*. food record, 24‐hour recall or food frequency questionnaire), including if and how portion sizes were assessed (*e.g*. using pictures, household measures, units or weighing). Indicate the administration method, purpose and population group for which the dietary assessment method was developed. In addition, describe how it was administered (*e.g*. by an interviewer, self‐ or proxy‐reported; face‐to‐face, by telephone, online or via mobile applications) and the steps taken to ensure quality of the assessment (*e.g*. training and supervision and/or data quality verification efforts). In case of food records and dietary recalls, indicate the number of days that were recorded or recalled, whether these were consecutive or non‐consecutive days and any specific characteristics of the food item, (*e.g*. low fat). Regarding food frequency questionnaires, report whether it was developed for any specific dietary component, the estimation of portion sizes, the time period covered and the number of food items included.

**nut‐8.2. Describe and justify food composition data used. Explain the procedure to match food composition with consumption data. Describe the use of conversion factors, if applicable**

If intake of nutrients or other components is calculated from the food consumption data, indicate the full source and justify the food composition data used. Give appropriate guidance (*e.g*. search strategy or references) if data are directly derived from peer‐reviewed publications. Report factors that influence the quality of the nutrient intake data, such as number of missing values in food composition data and how these were treated, how foods were matched, any conversion factors applied to the consumed food amounts (*e.g*. raw‐to‐cooked conversion) or food component concentrations (*e.g*. nutrient retention, yield or bioactivity).

**nut‐8.3. Describe the nutrient requirements, recommendations or dietary guidelines and the evaluation approach used to compare intake with the dietary reference values, if applicable**

If dietary intake data are evaluated against recommendations or reference values, report the authority and year of publication. Indicate the type of recommendations (*e.g*. adequate intake, average requirement, recommended dietary allowance, upper limit, dietary guideline or food‐based dietary guidelines) and their target group and describe the evaluation approach (*e.g*. probability method or cut‐point method) (Roman‐Vinas *et al*. 2009).

**nut‐8.4. When using nutritional biomarkers, additionally use the strobe extension for molecular epidemiology (STROBE‐ME). Report the type of biomarkers used and usefulness as dietary exposure markers**

When using nutritional biomarkers, report sample collection, processing, storage and analysis and use STROBE‐ME (Gallo *et al*. 2011). Report the validity and reliability of the biomarker as a marker of dietary exposure or nutritional status, including the time window for which the biomarker is representative.

**nut‐8.5. Describe the assessment of non‐dietary data (** ***e.g.*** **nutritional status and influencing factors) and timing of the assessment of these variables in relation to dietary assessment**

Describe the collection of non‐dietary data that could influence the estimates of dietary intakes. Include the time schedule for the collection of both dietary and non‐dietary data and the time period for each measurement in relation to each other.

**nut‐8.6. Report on the validity of the dietary or nutritional assessment methods and any internal or external validation used in the study, if applicable**

Describe and reference the validation study of the dietary or nutritional assessment method, including the reference method(s) used, when it was conducted, and in which population. The measures of validity should be reported (*e.g*. mean difference, correlation coefficient, classification agreement and limits of agreement), as well as its applicability at the individual level and the population level. Also report if the reproducibility has been tested.

**nut‐9. Report how bias in dietary or nutritional assessment (** ***e.g.*** **misreporting, changes in habits as a result of being measured, and data imputation from other sources) was addressed**

It should be clear how misreporting (including under‐ and over‐reporting) was defined and addressed in the analysis. Potential selection bias due to exclusion of misreporters should be assessed by comparing participant characteristics, and the potential influence on outcome should be discussed. Misreporting can arise as a result of poor recall of diet, interviewer bias or social acceptability bias. Similarly, bias such as regression to the mean should be considered.

**nut‐11. Explain the categorisation of dietary/nutritional data (** ***e.g.*** **use of N‐tiles and handling of non‐consumers) and the choice of reference category, if applicable**

Dietary intake data are often categorised in N‐tiles or in other categories (*e.g*. to express compliance with dietary recommendations). A clear description of the number of categories, cut‐off points and the choice of reference category is needed. The handling of non‐consumers during the analysis should be described to allow correct interpretation.

**nut‐12.1. Describe any statistical method used to combine dietary or nutritional data, if applicable**

Various methods can be used to combine food consumption and food composition data to estimate exposure through dietary intake of food groups, nutrients, other food components or contaminants. These methods (*e.g*. deterministic and the probabilistic approaches) should be clearly reported. If food and supplement intakes were combined, also report the method used.

**nut‐12.2. Describe and justify the method for energy adjustments, intake modelling and use of weighting factors, if applicable**

As intake of various nutrients and foods is associated with both energy and the intake of other nutrients or foods, adjustments may be needed to assess the diet–disease relationship (Willett 2013). Adjustment for total energy or energy from food can also mitigate the dietary assessment measurement error (Kipnis *et al*. 1997). Any adjustments and the methodology used should be clearly stated.
Report statistical techniques used to remove the within‐person error (*i.e*. when short‐term instruments such as 24‐hour dietary recalls are used to estimate the proportion of a population below or above a recommendation or cut‐off). Report weighting factors that might have been used, to ensure representativeness of seasons or the study population.

**nut‐12.3. Report any adjustments for measurement error (** ***i.e.*** **from a validity or calibration study)**

Even after using measurement error‐reduction techniques, dietary intake estimations may still be associated with substantial error. The overall magnitude of both random and systematic errors therefore needs to be considered in evaluation studies. If applicable, describe how the findings of reproducibility or validation studies undertaken were used to (partially) correct the observed results for measurement error.

**nut‐13. Report the number of individuals excluded based on missing, incomplete or implausible dietary/nutritional data**

Missing and implausible data are a pervasive problem in dietary investigations and may introduce bias or attenuated associations or lead to erroneous interpretations. Implausible data could be derived from incomplete dietary assessments or from (un)intentional under‐ or over‐reporting of dietary intake. Describe the number of missing values, cut‐offs for implausible data leading to exclusion, characteristics of those excluded, and any method used to handle missing values. Reporting of the number of individuals excluded will help to appraise the final power of the study and bias due to the exclusions.

**nut‐14. Give the distribution of participant characteristics across the exposure variables if applicable. Specify if food consumption of total population or consumers only were used to obtain results**

If relevant, the distribution of the participant characteristics (*e.g*. age, gender, lifestyle, health status and control/intervention groups) should be given according to the exposure variables. To allow correct interpretation, describe whether the distributions are based upon the total population or upon consumers only (*cf*. nut‐11). A visual representation of the distribution may facilitate the interpretation of findings.

**nut‐16. Specify if nutrient intakes are reported with or without inclusion of dietary supplement intake, if applicable**

Dietary supplements may contribute to the total intake of various nutrients. Failure to include these nutrient sources could lead to a serious underestimation of intake. To ensure correct interpretation and comparability of the findings, specify whether the nutrient intakes are derived from foods only or from both food and supplement intakes.

**nut‐17. Report any sensitivity analysis (** ***e.g.*** **exclusion of misreporters or outliers) and data imputation, if applicable**

Misreporters and outliers can be identified via several approaches [*e.g*. using cut‐offs (Goldberg *et al*. 1991) or N‐tiles]. If applicable, report any sensitivity analysis used to investigate the effect of data imputations or inclusion/exclusion of different population subgroups (*e.g*. misreporters or those that changed diets because of health reasons) on the study findings.

**nut‐19. Describe the main limitations of the data sources and assessment methods used and implications for the interpretation of the findings**

Dietary assessment methods are prone to various sources of bias and degrees of error that should be considered when interpreting the results. Limitations in food composition data should be described as well as the limitations inherent to the dietary assessment method (Slimani *et al*. 2015). Discuss whether the limitations could have introduced a random or systematic error and, if systematic, suggest in which direction this might have affected the findings.

**nut‐20. Report the nutritional relevance of the findings, given the complexity of diet or nutrition as an exposure**

Not all statistically significant findings are nutritionally relevant. Given the complexity of nutritional epidemiological research, over interpretation of findings should be avoided. Poor reporting of research findings may lead to implausible interpretations and spurious conclusions with regard to the relationship between nutrition and human health. If data allow, report effect sizes per serving with indications of weight or volume of the serving size to facilitate interpretation.

**nut‐22.1. Describe the procedure for consent and study approval from ethics committee(s)**

Ethical principles apply to nutritional research, and the procedures followed should be described. There are important differences between countries on the need to obtain approval of nutritional epidemiological studies by an appropriate committee or institution. Many nutritional journals currently request authors to comply with the appropriate procedure for ethical research and require describing the procedures followed during the study and for handling data.

**nut‐22.2. Provide data collection tools and data as online material or explain how they can be accessed**

Sharing of all research material is increasingly recognised as integral to good research practice. Sharing of data collection instruments such as questionnaires or software as online material contributes to the transparency of methods and findings. It enables reuse of instruments and may facilitate research to improve methods of dietary assessment and nutritional epidemiology. Similarly, access to food composition or participant level data allows reuse, independent (re)analysis, discovery and study replication. Machine‐readable formats are encouraged (Data citation synthesis group 2014). In case data cannot be shared publicly, researchers should explain this explicitly and provide clear instructions on how data can be accessed.

## Discussion

STROBE‐nut provides guidance for researchers to improve the quality and completeness of reporting in nutritional epidemiology. Although previous reporting guidelines have been proposed for use in dietary assessment (Nelson & Margetts 1997; FAO 2010; Burrows *et al*. 2012; Faber *et al*. 2013) or nutrition intervention studies (Welch *et al*. 2011), they were not developed following a consultative process to ensure broad consensus and support by their potential users. STROBE‐nut covered 94% of the recommendations for reporting of studies in these existing guidelines. Similar to other reporting guidelines, STROBE‐nut should not be used as a normative tool or a standard to appraise the quality of studies. STROBE‐nut complements the instructions of editorial and review processes to ensure a clear and transparent account of the research conducted.

Most experts contacted generally welcomed the initiative and provided constructive feedback. One expert did not see the value of research reporting guidelines in general, arguing that they only add a burden on the users. To address this, an assessment of the added value of developing additional guidelines such as STROBE‐nut will be conducted through a review of the use, effectiveness and user satisfaction of the STROBE‐nut checklist, organised 5 years after its first publication. In addition, collaboration with *Enhancing the Quality and Transparency of Health Research* (*EQUATOR*) network and STROBE will ensure complementarity with other reporting guidelines.

We will consider the STROBE‐nut guidelines in ongoing efforts that aim to add value in nutrition research, such as the *GloboDiet* initiative, the *European Nutrition Phenotype Assessment and Data Sharing Initiative* (*ENPADASI*) and the *Dietary Assessment Tool Network* (*DIET@NET*). We encourage prospective registration of protocols in public registries to increase the transparency of the research hypothesis, data analysis and completeness of reporting.

The STROBE‐nut guidelines are mainly geared towards reporting the methodological aspects of manuscripts. Although we do not present recommendations for writing the introduction of a study, it is clear that a critical assessment of the added value is needed to justify a study (Chalmers *et al*. 2014).

The structured and formal consultation process is a strength of STROBE‐nut. However, the dropout rate was substantial, and the response rate was lower compared to other reporting guidelines that used the Delphi technique (Tetzlaff *et al*. 2012; Vohra *et al*. 2015). The lower response rates are partly due to our efforts to consult as widely as possible, resulting in invitations for 200 experts. The final sample of experts that provided input, however, was adequate and still higher than most reporting guidelines that used face‐to‐face meetings or workshops (Simera *et al*. 2008). The increased participation towards the end and consensus on the final instrument makes us conclude that STROBE‐nut has satisfactory external support.

During the next years, the checklist will be translated and disseminated widely. Feedback through our website (www.strobe-nut.org) is encouraged to improve the checklist.

## Funding

There was no outside funding for this study.

## Competing Interests

The authors of this manuscript have the following competing interests: JEC is PI on a grant from the Medical Research Council (MR/L02019X/1) that is looking at ways to improve the quality of dietary assessment. MC acted as co‐ordinator of the STROBE collaboration. ME receives a stipend as a specialty consulting editor for PLOS Medicine and serves on the journal's editorial board.

## Conflict of Interest

The authors have no conflict of interest to disclose.

## Supporting information


**Table S1.** Responses received during the Delphi rounds.
